# Transmission of tuberculosis in an incarcerated population during the subclinical period: A cross-sectional study in Qingdao, China

**DOI:** 10.3389/fpubh.2023.1098519

**Published:** 2023-01-25

**Authors:** Zhongdong Wang, Haoran Li, Song Song, Haiyan Sun, Xiaoqi Dai, Meng Chen, Honghong Xu, Huaqiang Zhang, Yu Pang

**Affiliations:** ^1^Qingdao Municipal Center for Disease Control and Prevention, Qingdao, China; ^2^Department of Bacteriology and Immunology, Beijing Chest Hospital, Capital Medical University/Beijing Tuberculosis and Thoracic Tumor Research Institute, Beijing, China

**Keywords:** tuberculosis, incarcerated population, transmission, subclinical period, cross-sectional study

## Abstract

**Objectives:**

As a closed gathering place, prison is the cradle of tuberculosis (TB) outbreak. Therefore, the analysis of the prevalence rate and risk factors of latent tuberculosis infection (LTBI) in prison will be a necessary measure to intervene in the spread of tuberculosis.

**Methods:**

In this study, we consecutively recruited 506 adult prisoners in Qingdao to carry out this cross-sectional study. TB and LTBI were screened by IGRA, X-ray, X-pert, sputum smear and culture.

**Results:**

A total of 17 TB, 101 LTBI and 388 HC were identified, with an infection rate of 23.32% (118/506) and a TB incidence rate of 3282/100,000 population. Age, malnutrition and inmates living with TB prisoners were risk factors for LTBI. Additionally, most TB cases (70.59%, 12/17) were subclinical tuberculosis (STB), contributing significantly to TB transmission.

**Conclusion:**

Our results demonstrate that the transmission efficiency of asymptomatic patients is not essentially different from that of symptomatic patients, indicating that TB transmission occurs during the subclinical period. Our findings highlight the need to strengthen active case-finding strategies to increase TB case detection in this population.

## Introduction

*Mycobacterium tuberculosis* (MTB) is one of the leading causes of death due to a single infectious agent, accounting for an estimated 1.6 million death in 2021 (1). As an airborne-transmitted disease, MTB is majorly transmitted by inhaling droplet nuclei carrying viable bacilli. Although early clearance of MTB by innate immunity in the majority of cases, infection occurs in a substantial proportion of susceptible individuals, either leading to a dormant state of the bacilli or developing active tuberculosis (ATB) disease (2). It is estimated that approximately one-quarter of the world's population has been infected with tubercle bacilli (1). The LTBI is the major contributor to the pool of active tuberculosis cases, thereby constituting a pivotal barrier to TB elimination (3).

Prisons have consistently been recognized as high-risk environments for TB transmission due to overcrowded locations with poor ventilation (4). The estimated prevalence of infection in prisons is reported to be much higher than that in the general population (5). In a survey on TB control in Europe, it was estimated that European prions counted on average 17 times higher incidence than the general population (6). This is also true for low-income, high-burden countries, with more than 40 times higher TB prevalence rates in prions than in the general population (5). Given that infectious TB in prison inmates can be transmitted to the community after prisoners are released, prions have emerged as a threat to TB control; prisoners should be chosen as a priority population for intervention (7).

China has the second-highest TB burden in the world. Notably, the prevalence of bacteriologically positive TB exhibits significant geographic diversity across this country, varying from high in western China (212/100,000 population) to fewer than 66/100,000 population in eastern China (8). Qingdao is an eastern coastal city in China, with an incidence rate of 26.82/100,000 population, which is remarkably lower than the national average level (9). Despite significant achievements during past decades, prisons are neglected reservoirs of TB control in this region, majorly attributed to inadequate provision of health services (10). More importantly, international guidelines and national tuberculosis programmes focus on case detection and preventive interventions for certain high-risk groups (such as people living with HIV and family contacts with diagnosed TB patients), but relatively little attention is paid to incarcerated populations, thus hampering the formulation and implementation of effective policies for these individuals living in the risk environment (11). To address this concern, we undertook a cross-sectional study to determine the prevalence of active TB and risk factors of LTBI in a prison population.

## Methods

### Study design and participants

**Figure 1:**
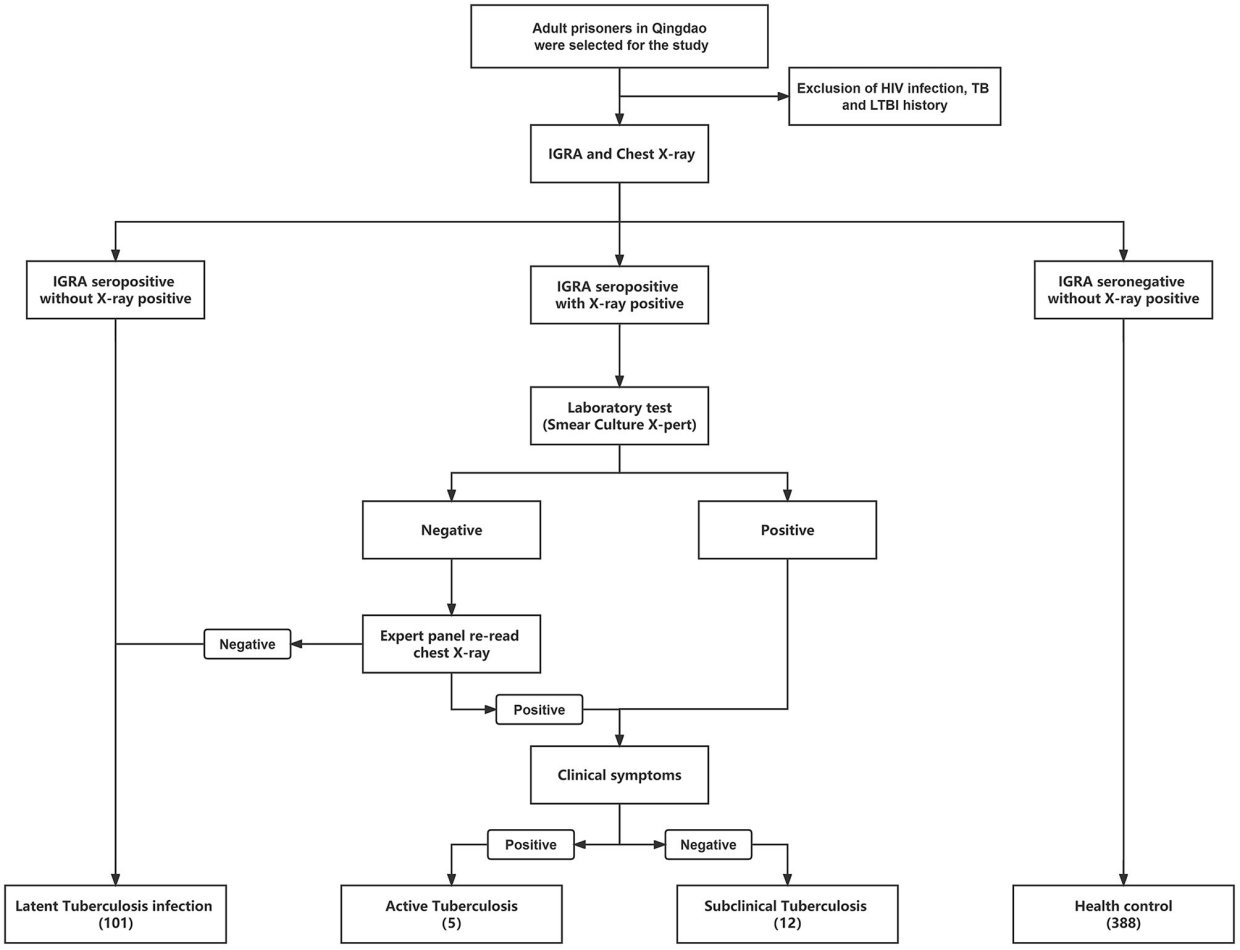
The workflow of this study is to classify different groups of prisoners with different health status. HIV, human immunodeficiency virus; TB, tuberculosis; LTBI, Latent Tuberculosis infection.

In 2022, we conducted cross-sectional screening for tuberculosis in prison in Qingdao, China. HIV/AIDS, TB and LTBI screening were carried out at the beginning of everyone's admission; HIV-positive, TB and LTBI prisoners were held incommunicado in different wards and excluded from this study. Finally, a total of 506 prisoners aged ≥18 were included. Demographic information was collected through a questionnaire, and preliminary TB screening was carried out. Further laboratory tests will be carried out on the screened TB patients (Figure 1).

### Ethical approval

This study was approved by the Ethics Commission of the Municipal Center of Disease Control and Prevention of Qingdao, and written informed consent was obtained from each participant before enrollment.

### TB screening

We collected the clinical symptoms of all participants. All participants underwent chest radiographs to diagnose tuberculosis in accordance with international standards and guidelines (8). Professional medical workers used BD Vacutainer Venous Blood Collection Tubes (Becton Dickinson, Sunnyvale, CA, USA) to collect venous blood while checked Mycobacterium bovis BCG vaccine scars.

The collected blood was centrifuged within 2 h at 2,500 rpm for 5 min. Then collect the upper serum, and next detected for IGRA through QuantiFERON-TB Gold Kit (QIAGEN, https://www.qiagen.com). Suspected TB (IGRA seropositive with X-ray positive) sputum samples were collected for smear, bacterial culture and X-pert detection ① Sputum smear was examined under a microscope after acid-fast staining. ≥1 acid-fast bacilli/100 visual fields were found in both examinations, which was defined as positive. ② The bacteria are cultured on Löwenstein–Jensen (L-J) medium, incubated at 37°C with daily examinations for 8 weeks until the sample isolates at most minuscule one *Mycobacterium tuberculosis* complex colony, which is defined as positive. ③ After adding the corresponding reagent of GeneXpert MTB/RIF (Xpert, Cepheid, Sunnyvale, CA, USA), the sample was processed according to the manufacturer's instructions, and finally put into the machine to read the results (12).

### Definitions

We defined the population of IGRA (–, negative) with X-ray (–) as Health control (HC); IGRA (–) with X-ray (+, positive) participants as LTBI. And performed a series of laboratory tests, including sputum smear, culture, and X-pert, on IGRA (+) with X-ray (+) participants. People who were positive in any of the laboratory tests were immediately defined as having TB, and the chest X-ray of all negative samples were re-read by medical imaging expert panel to determine whether it was TB (8). Patients with negative chest X-rays after re-read were included in the LTBI cohort. The positive population and the previous TB were defined as ATB and STB (**Table 3**), according to clinical symptoms (cough, expectoration, hemoptysis, etc.,).

### Statistical analysis

This study, the χ^2^ test was used to analyze the transition relationship between LTBI and HC. The risk factors from HC to LTBI were counted by multivariate logistic regression, and the corrected OR and 95% CI were calculated. The difference was considered statistically significant when *P* < 0.05. All calculations were performed using SPSS 22.0 software for Windows (SPSS Inc., Chicago, IL, United States).

## Result

### Study population

A total of 506 participants included 17 TB, 101 LTBI and 388 HC of which 17 TB were screened from 25 IGRA seropositive with X-ray positive participants using smear, culture, GeneXpert and rechecked chest X-ray, and a pulmonary TB incidence of 3,373 cases/100,000 persons (Table 1). Almost all of them were male (504/506, 99.60%), and only two females were LTBI and HC, respectively. The average age of the participants was 40 years old (range 18–78). Junior high school education accounted for 40.12% (203/506), followed by 38.34% (194/506) with high school education or above, and 21.54% (109/506) with primary school education. Most of the participants were non-local population, and only 15.42% (78/506) of the participants were local registered residents. 84.58% (428/506) of the participants were employed before being jailed. As of the time of information collection, the duration of incarceration was from 1 to 214 months (median term 2 months), and nearly half of the participants exceeded 2 months in jail (262/506, 51.78%).

**Table 1:** Comparison of characteristics in prisoners, Qingdao, China.

**Characteristics**	**Total**	**TB**	**LTBI**	**HC**	***P*-value by χ^2^ test**
	***n* = 506** **(100.0%)**	***n* = 17** **(3.36%)**	***n* = 101** **(19.96%)**	***n* = 388** **(76.68%)**	
**Sex**					
Male	504 (99.60%)	17 (100.00%)	100 (99.01%)	387 (99.74%)	0.304
Female	2 (0.40%)	0 (0.00%)	1 (0.99%)	1 (0.26%)	
**Age (years)**					
< 25	39 (7.71%)	4 (23.53%)	4 (3.96%)	31 (7.99%)	0.049
25–34	154 (30.43%)	6 (35.29%)	26 (25.74%)	122 (31.44%)	
35–44	141 (27.87%)	3 (17.65%)	25 (24.75%)	113 (29.12%)	
≥45	172 (33.99%)	4 (23.53%)	46 (45.54%)	122 (31.44%)	
**Education level**					
Primary school	109 (22.33%)	4 (23.53%)	28 (27.72%)	77 (19.85%)	0.229
Middle school	203 (41.11%)	4 (23.53%)	38 (37.62%)	161 (41.49%)	
High school	194 (38.74%)	9 (52.94%)	35 (34.65%)	150 (38.66%)	
**Residence**					
Local	364 (71.94%)	13 (76.47%)	66 (65.35%)	285 (73.45%)	0.107
Migrant	142 (28.06%)	4 (23.53%)	35 (34.65%)	103 (26.55%)	
**Employment**					
No	78 (15.42%)	3 (17.65%)	21 (20.79%)	54 (13.92%)	0.088
Yes	428 (84.58%)	14 (82.35%)	80 (79.21%)	334 (86.08%)	
**Malnutrition (BMI**<**18.5)**					
No	440 (86.96%)	14 (82.35%)	94 (93.07%)	332 (85.57%)	0.045
Yes	66 (13.04%)	3 (17.65%)	7 (6.93%)	56 (14.43%)	
**Contact history**					
No	431 (85.18%)	13 (76.47%)	77 (76.24%)	341 (87.89%)	0.003
Yes	75 (14.82%)	4 (23.53%)	24 (23.76%)	47 (12.11%)	
**Concurrent condition**					
No	408 (80.63%)	14 (82.35%)	76 (75.25%)	318 (81.96%)	0.430
Yes	98 (19.37%)	3 (17.65%)	25 (24.75%)	70 (18.04%)	
***Mycobacterium bovis*** **BCG scar**					
No	452 (89.33%)	15 (88.24%)	89 (88.12%)	348 (89.69%)	0.648
Yes	54 (10.67%)	2 (11.76%)	12 (11.88%)	40 (10.31%)	
**Current duration of incarceration, month**					
< 2	244 (48.22%)	2 (11.76%)	52 (51.49%)	190 (48.97%)	0.652
≥2	262 (51.78%)	15 (88.24%)	49 (48.51%)	198 (51.03%)	
**Living in the same room as TB inmates**					
No	351 (69.37%)	0 (0.00%)	18 (17.82%)	333 (85.82%)	<0.001
Yes	155 (30.63%)	17 (100.00%)	83 (82.18%)	55 (14.18%)	

TB, tuberculosis; LTBI, Latent tuberculosis infection; HC, Health control; BCG, Bacillus Calmette Guerin; BMI, body mass index; OR, odds ratio; CI, confidence interval.

According to the WHO standard, 13.04% (66/506) of people were malnutrition (BMI <18.5) (13, 14). 15.61% (79/506) of the participants had a history of close contact with TB (family member with a TB history and history of close contact with a TB patient). Surprisingly, only 10.67% (54/506) of people with the BCG vaccine (*Mycobacterium bovis* BCG scar). 19.37% (98/506) of participants had complications. After screening, 30.63% (155/506) of them were found to share a room with TB inmates.

### Characteristics between LTBI and HC

Preliminary tests showed that 19.96% (101/506) of the prisoners changed from HC to LTBI during their incarceration. Therefore, we compared the characteristic frequencies of LTBI and HC (Table 2). In terms of age, most people with LTBI were ≥45 years old (45.54%, 46/101). The IGRA-seropositive was proportional to age, and the risk of becoming LTBI increases (*P* < 0.05). In addition, malnourished prisoners were at greater risk and had a higher incidence (*P* = 0.045, 95% CI: 1.024–7.091). Remarkably, there was a significant relationship between the high incidence of LTBI and the cohabitation of the TB population we had identified (*P* < 0.001, up to 50–60 people per cell), and it was an inextricable risk factor (*P* < 0.001, 95% CI: 0.010–0.042).

**Table 2:** Factors associated with LTBI among prisoners, Qingdao, China.

**Factor**	**OR (95% CI)**	***P*-value by** **multivariable regression**
**Age (years)**		
< 25	Referent	0.044
25–34	4.809 (1.309–17.665)	
35–44	1.759 (0.869–3.563)	
≥45	2.110 (1.031–4.318)	
**Malnutrition (BMI**<**18.5)**		
No	Referent	0.045
Yes	2.694 (1.024–7.091)	
**Contact history**		
No	Referent	0.892
Yes	0.952 (0.467–1.939)	
**Living in the same room as TB inmates**		
No	Referent	<0.001
Yes	0.952 (0.467–1.939)	

TB, tuberculosis; LTBI, Latent tuberculosis infection; OR, odds ratio; CI, confidence interval.

In addition, the sentence length and place of origin were not related to the incidence of LTBI in this study (*P* = 0.652, 0.107). Participants with a history of close contact with TB had a higher incidence (*P* =0.003), but did not affect the conversion of HC to LTBI (*P* = 0.892, 95% CI: 0.467–1.939). However, the incidence of LTBI was not statistically significant in terms of education level, pre-prison work, excess nutrition, complications, and BCG vaccination (*P* > 0.05).

### Subclinical tuberculosis transmission ability

**Figure 2:**
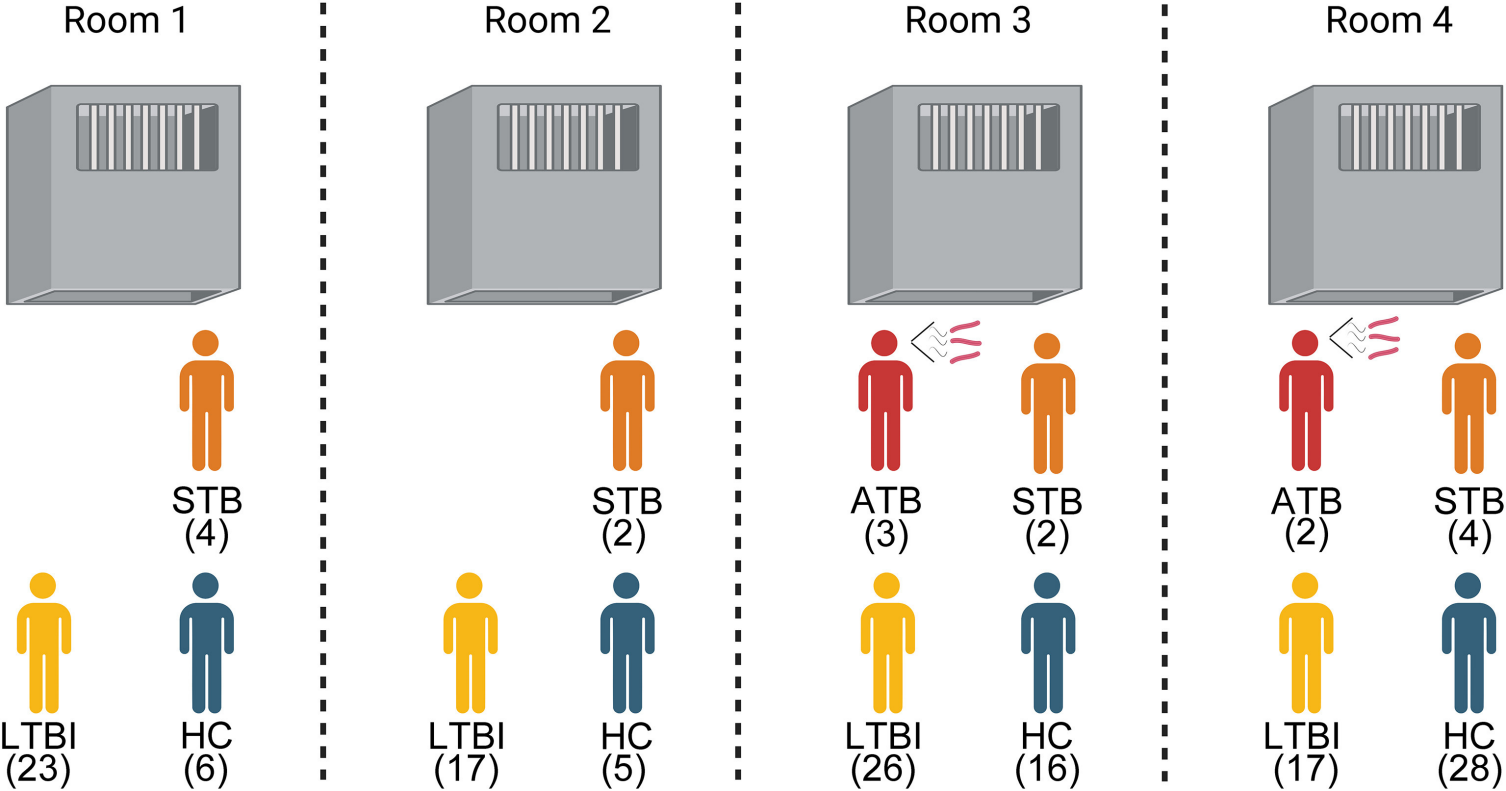
Composition of the health status of prisoners living with tuberculosis. ATB, Active tuberculosis; STB, Subclinical tuberculosis; LTBI, Latent Tuberculosis infection; HC, Healthy control.

We further improved the demographic, clinical symptoms and laboratory testing information of 17 TB (Table 3). It was found that this TB was mainly concentrated in four cells. And 70.59% (12/17) of TB were in the subclinical tuberculosis (STB) state; there were no clinical symptoms of tuberculosis such as cough and expectoration. Even half of the cells had only STB prisoners, but it still caused HC in the same room to be infected with LTBI (Figure 2). This indicated that STB still has a solid ability to spread tuberculosis.

**Table 3:** Information of prisoners with tuberculosis.

**Patient ID**	**Room number**	**Demographic information**				**Clinical symptoms**								**Laboratory testing**		
		**Age**	**Sex**	**Employment**	**Resistance**	**Cough**	**Cough up phlegm**	**Hemoptysis**	**Chest pain**	**Night sweat**	**Weakness**	**Smoking history**	**History of tuberculosis**	**Smear**	**Culture**	**Xpert**
1	1	41	M	•	•	◦	◦	◦	◦	◦	•	•	◦	◦	◦	◦
2		23	M	•	◦	◦	◦	◦	◦	◦	•	◦	◦	◦	◦	◦
3		28	M	•	•	◦	◦	◦	◦	◦	•	•	◦	◦	◦	◦
4		32	M	•	•	◦	◦	◦	◦	◦	•	◦	◦	◦	◦	•
5	2	25	M	◦	◦	◦	◦	◦	◦	◦	◦	•	•	◦	◦	◦
6		31	M	•	•	◦	◦	◦	◦	◦	◦	◦	◦	◦	◦	•
7	3	60	M	•	◦	•	◦	◦	◦	◦	◦	◦	◦	◦	◦	•
8		33	M	•	•	•	◦	◦	◦	◦	◦	◦	◦	◦	◦	◦
9		18	M	•	•	•	◦	◦	◦	◦	◦	◦	◦	◦	◦	•
10		43	M	•	•	◦	◦	◦	◦	◦	•	◦	•	◦	◦	◦
11		42	M	•	•	◦	◦	◦	◦	◦	◦	•	◦	◦	•	•
12	4	53	M	◦	•	◦	◦	◦	◦	◦	◦	•	◦	◦	◦	•
13		23	M	•	•	◦	◦	◦	◦	◦	◦	◦	◦	◦	•	•
14		21	M	◦	•	•	◦	◦	◦	◦	•	◦	◦	◦	•	•
15		56	M	•	•	•	◦	◦	◦	◦	◦	•	•	◦	◦	◦
16		45	M	•	◦	◦	◦	◦	◦	◦	◦	◦	◦	◦	◦	◦
17		28	M	•	•	◦	◦	◦	◦	◦	◦	◦	◦	◦	◦	◦

◦ Indicate no/negative; • Indicate yes/positive; M, male.

## Discussion

Prisons are regarded as a hotspot for TB disease and transmission, emphasizing that prevention strategy must focus on this hotspot to achieve the global target of ending TB (11). In the present study, we confirm the prediction of a significant burden of TB in an incarcerated population in Qingdao, where 3.36% of inmates had pulmonary TB. The prevalence of TB found in our study was six times higher than the estimated prevalence for the general population (0.5% in 2010) (8). This prevalence corroborated that in Cameroon (3.3%) (5) and Zambia (4.0%) (15), although it is lower than that in Ethiopia (10.4%) (16), and South Africa (7.5%) (17), and higher than that in Malawi (0.7%) (18). The cause for this discrepancy is majorly due to different sampling strategies, screening methodologies, and case definitions across studies (17). Indeed, several of the studies mentioned above registered only prisoners with typical symptoms and chest radiographs as having smear-positive pulmonary TB, which leads to a possible underestimation of the rate of pulmonary TB. The high prevalence of active TB in incarcerated populations calls for urgent strategies to address this concern, considering that it is not only a risk to other prisoners and prison staff; it also has the potential to put the communities in danger of TB disease after their release from prison.

By employing the active case-finding strategy, 17 cases of active pulmonary TB were detected in our cohort, of which 5 had clinical symptoms suggestive of active TB and 12 were declared free of symptoms, demonstrating that about two-thirds of patients were subclinical diseases. By reviewing data from TB prevalence population surveys, Frascella and colleagues found that the median percentage of subclinical TB cases was 50.4 and 56.4% in African and Asian countries, respectively (19). Existing data are increasingly clear that subclinical TB accounts for a substantial fraction of prevalent disease in the general population, as well as in the incarcerated population, suggesting the heterogeneity in the natural history of TB. In the subclinical period, the absence of symptoms indicating active TB challenges the passive case-finding strategy for diagnosing TB in most low-income countries, where chest radiography is conducted only for cough of 2 or more weeks' duration, undoubtedly resulting in subclinical TB patients without clinical symptoms are missed. This could be intensified by the fact that prisons are often underfunded and without mandate to provide comprehensive healthcare services (7). Thus, our findings highlight the need to strengthen active case-finding strategies to increase TB case detection in this population.

Our finding of a great proportion of subclinical TB inmates raised the very interesting question of whether TB transmission occurred during the subclinical period. Traditionally, it is well known that TB transmission is caused by the aerosolization of infectious droplet nuclei, which are majorly generated by coughing (20). A previous household study in Uganda demonstrated that cough-expelled aerosols with tubercle bacilli were a much stronger predictor of recent infection (21). However, our results supported the opposite notion, that the transmission efficiency of asymptomatic patients was not essentially different from that of symptomatic patients. One possible explanation for the infectivity of asymptomatic patients is that respiratory droplets can produce during routine activities such as talking and breathing (22). In line with our hypothesis, multiple recent studies by analyzing MTB DNA from face masks worn by individuals with active TB found no association between the quantity of exhaled MTB and cough frequency or sputum positivity (23). Despite exhibiting no disease symptoms, people can still have varying bacterial loads contributing to further transmission. Similar results were reported in COVID-19-infected patients that symptom expression was not always positively correlated with virus load. This phenomenon may reflect that clinical symptoms are determined by an intricate balance between the immune system of the host and the virulence of tubercle bacilli. It also underlines the fact that the majority of individuals are highly susceptible to TB because of the limited protective efficacy of the BCG vaccine; thus infection with MTB can occur even after exposure to small numbers of viable bacilli (23).

We also explored potential risk factors for TB infection in an incarcerated population. As expected, the inmates exposed to hospital roommates with active TB disease were the most significant factor for TB infection. This could be explained by the nature of the cells shared by the inmates, including high-density crowds and poor ventilation. In addition, we found a trend toward low BMI being associated with an increased risk of TB infection. An observational study concerning the influence of BMI on immune response indicated that low BMI was related to the impairment of cell-mediated immunity functions (24), which is essential for MTB clearance in host. Moreover, Han and colleagues found that white adipose tissue provided a niche for the long-term maintenance of pathogen-specific memory T cells (25). Therefore, impaired T cell immunity is a potential major contributor to an increased risk of TB infection in individuals with low BMI.

This study had several limitations. First, the major limitation of the present study was the small number of inmates, which may limit the overall significance of our conclusion. Second, due to the cross-sectional nature of this study, we did not conduct intensive follow-ups on individuals with evidence of recent infection. Third, a recent modeling study demonstrated that the sensitivity of chest X-rays for asymptomatic individuals might be lower than 88% (23), indicating that the burden of subclinical TB in our cohort may be underestimated. In addition, further laboratory testing of the cases with IGRA or CXR negative cohorts may help to find hidden TB or LTBI. Finally, only a small number of patients had positive cultures, hampering us to perform molecular genotyping analysis, and identifying the timing and directionality of disease transmission.

To conclude, we confirm the prediction of a large burden of TB in an incarcerated population in Qingdao, where 3.3% of inmates had pulmonary TB. About two-thirds of patients are subclinical diseases without symptoms indicating active TB. In addition, our results demonstrate that the transmission efficiency of asymptomatic patients is not essentially different from that of symptomatic patients, indicating that TB transmission occurs during the subclinical period. The inmates exposed to hospital roommates with active TB disease and low BMI are great risk factors for TB infection. Our findings highlight the need to strengthen active case-finding strategies to increase TB case detection in this population.

## Data availability statement

The original contributions presented in the study are included in the article/supplementary material, further inquiries can be directed to the corresponding authors.

## Ethics statement

This study was approved by the Ethics Commission of Municipal Center of Disease Control and Prevention of Qingdao and written informed consent was obtained from each participant prior to enrollment. The patients/participants provided their written informed consent to participate in this study.

## Author contributions

YP and ZW: conceptualization and methodology. ZW, HL, MC, and SS: formal analysis and investigation. XD, SS, MC, and HS: data curation. ZW and HL: writing—original draft preparation. YP and HZ: writing—review and editing. YP: funding acquisition. All authors contributed to the article and approved the submitted version.
